# The reticulocyte restriction: invasion ligand RBP1a of *Plasmodium vivax* targets human TfR1, prohibitin-2, and basigin

**DOI:** 10.3389/fcimb.2025.1671048

**Published:** 2025-09-25

**Authors:** Jessica Molina-Franky, Daniel Röth, Monica Ararat‐Sarria, Manuel Alfonso Patarroyo, Markus Kalkum

**Affiliations:** ^1^ Department of Immunology and Theranostics, Arthur Riggs Diabetes and Metabolism Research Institute, Beckman Research Institute of the City of Hope, Duarte, CA, United States; ^2^ Global Scholars Program, Beckman Research Institute of the City of Hope, Duarte, CA, United States; ^3^ Grupo de Investigación Básica en Biología Molecular e Inmunología (GIBBMI), Fundación Instituto de Inmunología de Colombia (FIDIC), Bogotá, Colombia; ^4^ PhD Program in Biotechnology, Universidad Nacional de Colombia, Bogotá, Colombia; ^5^ School of Medicine and Health Sciences, Universidad del Rosario, Bogotá, Colombia; ^6^ Microbiology Department, Faculty of Medicine, Universidad Nacional de Colombia, Bogotá, Colombia

**Keywords:** *Plasmodium vivax*, parasite invasion, erythroid cell lines, membrane proteomics, receptor-ligand interactions, LC-MS proteomics

## Abstract

**Introduction:**

*Plasmodium vivax* is the most widespread cause of malaria outside Africa. Developing effective controls is challenging because *P. vivax* exclusively invades reticulocytes, immature erythrocytes that are scarce and short-lived. This limits opportunities to culture the parasite and investigate the receptor-ligand interactions crucial for host cell invasion.

**Methods:**

The erythroid cell lines JK-1 and BEL-A were evaluated *in vitro* as reticulocyte surrogates to assess their susceptibility to *P. vivax* invasion. Comparative membrane proteomics of these cell lines, reticulocytes, and mature erythrocytes were performed using quantitative liquid chromatography–mass spectrometry (LC-MS). Specific interactions between the parasite ligand PvRBP1a (residues 158–650) and candidate host receptors were identified by TurboID proximity labeling and validated through ELISA binding assays.

**Results:**

We confirmed that the JK-1 cell line supports *P. vivax* invasion and demonstrated for the first time that BEL-A cells are similarly susceptible, establishing both as effective surrogate models. Membrane proteomics identified several receptor candidates potentially involved in selective host-cell entry. In addition to known receptors, including transferrin receptor protein 1 (TfR1/CD71), CD98hc, and basigin (BSG), novel receptor candidates such as prohibitin-2 (PHB2), CAT-1 (SLC7A1), ATB(0) (SLC1A5), CD36, integrin beta-1 (ITGB1), and metal transporter CNNM3 were discovered. Proximity labeling with a recombinant PvRBP1a (158–650)-TurboID fusion protein confirmed the known interactions with TfR1 and BSG, and additionally identified PHB2 as a novel interacting partner. Notably, this is the first report implicating PHB2 as a co-receptor for *P. vivax* invasion.

**Conclusion:**

Our findings provide novel insights into the molecular mechanisms underlying reticulocyte restriction in *P. vivax*. The JK-1 and BEL-A cell lines represent valuable platforms for dissecting receptor–ligand interactions during parasite invasion and for advancing the development of targeted therapeutic antimalarial strategies.

## 1 Introduction

Malaria remains a major global health issue, with 263 million cases reported in 2023. *Plasmodium vivax*, one of the six human-infecting *Plasmodium* species, is noteworthy for its global distribution. In the Americas, most malaria cases are due to *P. vivax* (72.1% in 2023) (World Health Organization, 2024). Its blood stage form, the merozoites, interact with red blood cell (RBC) receptors leading to RBC invasion (Gruszczyk et al., 2018a; Malleret et al., 2021; Molina-Franky et al., 2022). However, *P. vivax* exclusively infects reticulocytes (Malleret et al., 2015a), immature, short-lived precursors of erythrocytes, posing a significant challenge to research progress. In contrast, *P. falciparum* has been extensively researched due to the availability of a well-established *in vitro* culture system for over 40 years (Trager and Jensen, 1976). This critical disparity highlights the urgent need to develop alternative research models for *P. vivax*.

The mechanism of *P. vivax* reticulocyte invasion remains unclear. It was previously believed that *P. vivax* exclusively targeted reticulocytes through the Duffy antigen receptor for chemokines (DARC) and Duffy binding protein (PvDBP) interaction (Horuk et al., 1993), as individuals with the Fy(a-b-) mutation in West Africa were resistant to the infection. However, DARC is present on both reticulocytes and erythrocytes, and *P. vivax* infections have been documented in Duffy-negative populations (Ryan et al., 2006; Reyes et al., 2022; Picón-Jaimes et al., 2023).

Together with evidence from Duffy-negative infections, geographical variation among *P. vivax* isolates further supports the idea that invasion is not limited to a single pathway. Transcriptomic studies show that invasion-related genes such as *PvRBP1a*, *PvRBP2a*, and *PvRBP2b* are more highly expressed in Ethiopian and Cambodian isolates than in Brazilian isolates, while *PvDBP1* and *PvEBP/DBP2* are elevated in Cambodian parasites. These patterns suggest that *P. vivax* employs multiple, regionally adapted invasion strategies (Kepple et al., 2023).Among the most prominent candidates are the reticulocyte-binding protein (RBP) family, which may interact with transferrin receptor 1 (TfR1) and CD98 heavy chain (SLC3A2), both of which are lost during the maturation of reticulocytes to erythrocytes (Galinski et al., 1992; Gruszczyk et al., 2018a; Malleret et al., 2021). This implies that *P. vivax* (Pv)RBP family proteins specifically target receptors unique to the reticulocyte membrane. Our previous studies on PvRBP1 of the *P. vivax* strain Belem (GenBank AAA29743.3) identified eleven high-affinity reticulocyte binding peptides (HABPs) corresponding to residues 158–653 of PvRBP1a in the *P. vivax* Salvador I strain (GenBank AAS85749.1). Among these, HABP 3742 (KLLGEEISEVSHLYV) and HABP 3459 (KEILDKMAKKVHYLK) exhibited dissociation constants (Kd) of 131 nM and 155 nM, respectively (Urquiza et al., 2002). Additionally, an extracellular portion of PvRBP1a, residues 157-650, binds strongly (~50%) to reticulocytes and moderately (~20%) to erythrocytes (Ntumngia et al., 2018). The identity of PvRBP1a_157–650_ binding-receptors within the reticulocyte membrane has been unclear. Therefore, this study evaluated the erythroleukemic cell line JK-1 (Okuno et al., n.d) and the Bristol Erythroid Line Adult (BEL-A) (Trakarnsanga et al., 2017) as surrogates for reticulocytes, examining their susceptibility to *P. vivax* invasion. A comparison of the cell lines’ membrane proteomes revealed similarities with those of reticulocytes, and dissimilarities with erythrocyte membrane proteomes, thereby identifying potential *P. vivax* receptors. Furthermore, TurboID proximity labelling implied specific interactions of PvRBP1a_158–650_ with prohibitin-2 (PHB2), TfR1, and basigin (BSG). These interactions were confirmed by ELISA, highlighting key molecular determinants of *P. vivax’s* reticulocyte tropism.

## 2 Materials and methods

### 2.1 Ethics statement

The study was conducted in accordance with the Declaration of Helsinki. Use of anonymized discarded blood from therapeutic phlebotomy was approved by the Institutional Review Board of City of Hope, Duarte, California, USA, as exempt category 4, under 45CFR46.104 (d). Blood samples from malaria patients were obtained under informed consent with the approval of the Bioethics central committee of the Universidad de Córdoba, Monteria, Colombia, and imported into the United States under CDC permit No.: 20210830-3188A0.

### 2.2 Collection, processing, and enrichment of *P. vivax* parasites from blood samples

**Figure 1:**
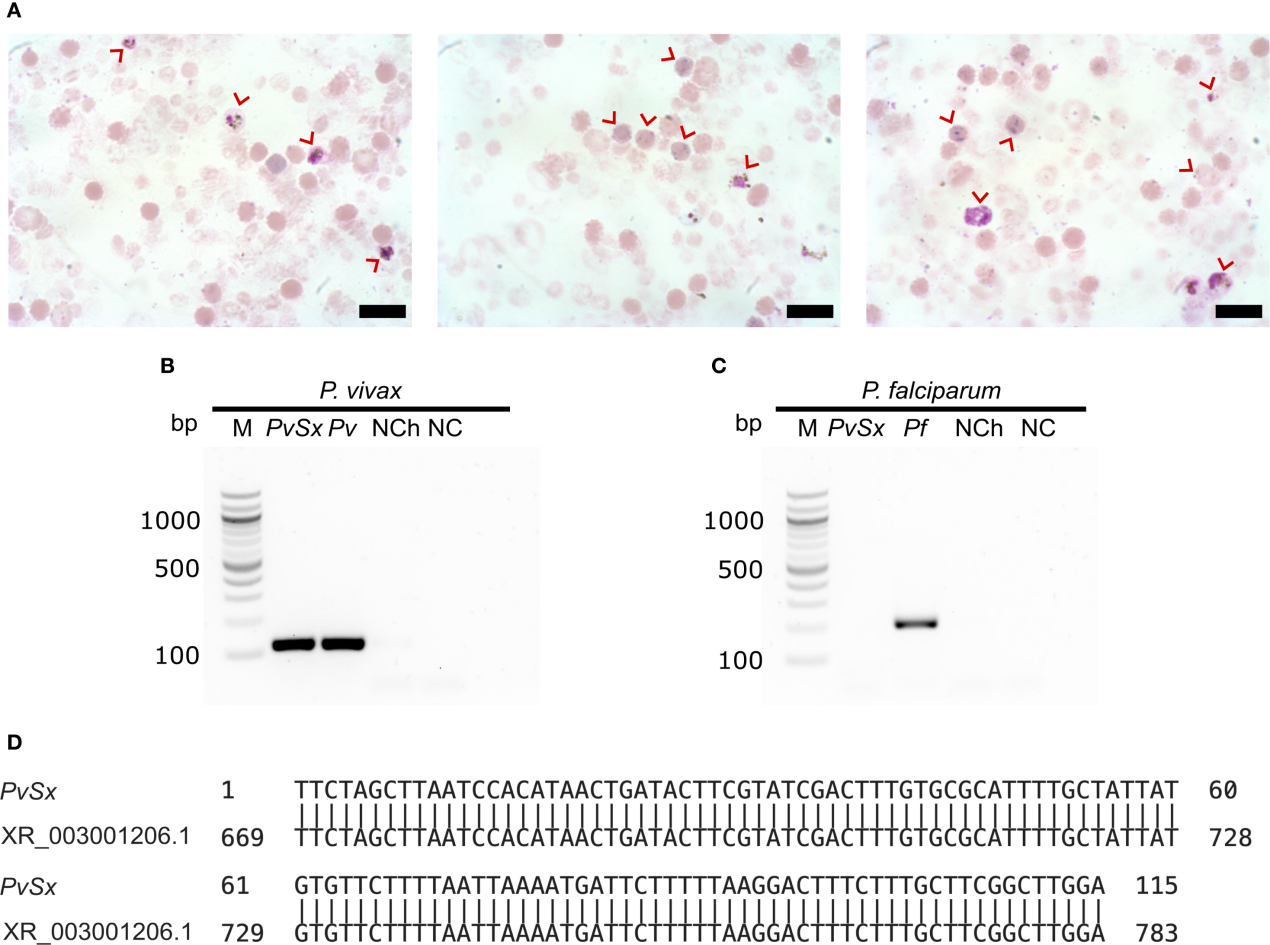
P*. vivax* mono-infected reticulocytes. **(A)** Three representative micrographs of *P. vivax*-infected enriched reticulocytes (red arrows) from a malaria patient. Scale bars are 3 µm. **(B, C)** Species-specific nested PCR for the small subunit 18S ribosomal RNA. Lane M, 100 bp marker; Lane PvSx, sample used in the infection of erythroid cells; Lanes Pv and Pf belong to the positive controls for each species; lane NCh, negative control using genomic DNA from a healthy human; lane NC, negative control using water instead of genomic DNA. **(B)** *P. vivax* (∼120 bp amplicon). **(C)** *P. falciparum* (∼205 bp amplicon). **(D)** DNA sequence alignment of the positive PvSx amplicon with the corresponding gene segment of the *P. vivax* Salvador-1 reference strain (GenBank No. XR_003001206.1) (Carlton et al., 2008), showing 100% identity.

*P. vivax* infected blood samples were collected from malaria patients in Tierralta—Córdoba, Colombia, into 5-mL sodium citrate tubes. After transportation to Bogotá, RBCs were enriched by centrifugation, mixed with an equal volume of Glycerolyte 57, cryopreserved, shipped to the U.S. lab, thawed using the NaCl method (Blomqvist, 2008), and resuspended in 3 mL of Iscove’s Modified Dulbecco’s Medium (IMDM). These RBCs were then enriched from 0.2% to 4.0% parasitemia by concentrating *P. vivax*-infected reticulocytes through a KCl-Percoll gradient (Rangel et al., 2018). Enrichment was evaluated by microscopy with Giemsa staining (**Figure 1A**).

### 2.3 Identification of the *Plasmodium* species

Genomic DNA was extracted from infected cells, and nested PCR was performed using this DNA to identify the *Plasmodium* species. Genus- and species-specific primers targeting the parasite’s 18S ribosomal small subunit RNA were used as previously described (Snounou et al., 1993) (see **Supplementary Data**).

### 2.4 *P. vivax* entry into BEL-A and JK-1 cells

JK-1 cells were obtained from the *Deutsche Sammlung von Mikroorganismen und Zellkulturen GmbH* (DSMZ) in Braunschweig, Germany, and BEL-A cells were from Prof. Dr. Jan Frayne of the University of Bristol, under contract by the NHS Blood and Transplant of the UK, see acknowledgement for details.

The enriched *P. vivax* mono-infected RBC sample (~40 µL) was divided into two equal aliquots, one for co-incubation with BEL-A cells and the other with JK-1 cells. Each cell line (1.5x105 cells) was cultured in 500 µL of medium, for BEL-A in StemSpan serum-free expansion medium (SFEM, StemCell Technologies), containing 25% human serum (Type AB, Sigma-Aldrich), 50 ng/mL stem cell factor (SCF), 3 U/mL erythropoietin (EPO), 1 μM dexamethasone, 1 μg/mL doxycycline, 1 μg/mL chemically defined lipid concentrate (CDLC), and 100 µM hypoxanthine; and JK-1 in IMDM supplemented with GlutaMAX, with the same components, except for SCF, EPO, dexamethasone, and doxycycline. Both cultures were incubated at 37 °C with 5% CO2 and 5% O2. Fresh medium and 2×105 erythroid cells were added every two days, and cultures were evaluated by immunofluorescence assay (IFA) every 24 hours for 6 days (see **Supplementary Data**).

### 2.5 Quantitative comparison of membrane proteomes by (DIA) - LC-MS/MS

Reticulocytes, erythrocytes, JK-1 and BEL-A cells were collected, cytoplasmic content was removed by osmolytic lysis, and membrane proteins of the remaining ghosts were extracted. From each sample, 23 μg of proteins were processed for proteomics using S-Trap columns (ProtiFi) according to the manufacturer instructions (Matulis, 2016; HaileMariam et al., 2018). The resulting trypsin/LysC-digested peptides were analyzed by LC-MS/MS in data-independent acquisition (DIA) mode, as detailed in the **Supplementary Data**.

To enrich the plasma membrane proteins from the data set, proteins were filtered based on at least one of the following annotations from the UniProt subcellular localization database: “Cell membrane”, “Apical cell membrane”, “Basolateral cell membrane”, “Peripheral membrane protein” and “Plasma membrane”, and the Gene Ontology (GO) annotation term: “plasma membrane”. The topology of the membrane proteins abundant in reticulocytes compared to erythrocytes was evaluated using several predictors. Protein sequences were analyzed with Protter for overall visualization of proteoforms (Omasits et al., 2014), TMHMM 2.0 for transmembrane region prediction (Hallgren et al., 2022), SignalP 6.0 for signal peptide identification (Teufel et al., 2022), and PredGPI for GPI anchor site prediction (Pierleoni et al., 2008).

### 2.6 PvRBP1A_158–650_ proximity labeling for the identification of likely receptor candidates

#### 2.6.1 Cloning, expression, purification, and activity of TurboID fusion proteins

The DNA sequence encoding PvRBP1a_158-650_ (GenBank AAS85749.1) was derived from the *P. vivax* Salvador I reference strain (txid126793). This sequence was fused to an acidic linker (L), GDEVDEDEG, to improve solubility, and the TurboID protein (TID) (Branon et al., 2018), followed by a C-terminal 6xHis tag for purification, resulting in the PvRBP1a_158-650_LTID fusion protein. An equivalent gene encoding L with TurboID alone (LTID) was designed as a negative control. Both gene constructs were obtained as customized synthetic genes, optimized for expression in *E. coli*, and cloned into a pET-28a(+) expression vector between its NcoI and XhoI sites. The constructs were expressed in soluble form in *E. coli* BL21 cells and purified by affinity chromatography, as detailed in the **Supplementary Data**. Protein purity and expression were verified by polyacrylamide gel electrophoresis. The biotinylation activity of both recombinant TurboID fusion proteins was confirmed by evaluating their autobiotinylation activity through incubation in the presence or absence of biotinylation reaction buffer Brxn (20 mM Tris-HCl, 500 µM biotin, 2.5 mM ATP, pH 7.5) at 37 °C for 15 minutes, quenching on ice and Western blot analysis with streptavidin-IRDye 800CW conjugate (1:1,000) and an Odyssey DLx imaging system (LICORbio).

#### 2.6.2 PvRBP1a_158-650_LTID proximity labeling

To evaluate PvRBP1a_158-650_LTID’s interaction with JK-1, BEL-A, reticulocytes, and erythrocytes, proximity labeling assays were performed in duplicate, and repeated up to three times. Cells were washed twice with PBS supplemented with 2% human serum (HS 2%) and incubated with either PvRBP1a_158-650_LTID or LTID (negative control) for 3 hours at room temperature with constant mild agitation at 10 rpm. Following incubation, cells were washed three times with HS 2% to remove unbound proteins, then incubated with Brxn for 15 minutes at 37°C. The reaction was stopped by cooling on ice for 5 minutes, and the samples were washed with cold HS 2% before labeling with Alexa Fluor 488-conjugated streptavidin (10 µg/mL, Invitrogen) for 1 hour at room temperature. Biotinylation was quantified by cytometry, acquiring 100,000 events per sample on a FACSAria Fusion (BD). Data were analyzed with FlowJo v10.8.1 (Ashland et al., 2023), calculating the percentage of biotinylated cells relative to total cells. LTID-treated and unlabeled cells served as negative controls.

#### 2.6.3 The biochemical nature of PvRBP1a_158–650_ receptors

JK-1 and BEL-A cells were treated with trypsin (1 mg/mL, Sigma-Aldrich), chymotrypsin (1 mg/mL, Sigma-Aldrich), or neuraminidase (50 mU, Roche) for 1 hour. After enzymatic treatment, proteolytic enzymes were inactivated with soy trypsin inhibitor (0.5 mg/mL, Sigma-Gibco) (Deans et al., 2007). Proximity labeling assays were then performed as described above, using PvRBP1a_158-650_LTID or LTID (3 µM).

#### 2.6.4 Affinity enrichment and LC-MS identification of PvRBP1a_158–650_ proximity-labeled receptor candidates

JK-1 cells were incubated with either PvRBP1a_158-650_LTID or LTID (negative control), each at 3 µM for 3 hours. After incubation, cells were washed three times with HS 2%, then incubated with 100 µL of Brxn for 15 minutes at 37°C. The reaction was stopped as described above, and cells were resuspended in 500 µL of IP-MS lysis buffer (MS-compatible Magnetic IP kit, streptavidin, Pierce, Thermo Scientific), incubated on ice for 30 minutes with intermittent vortexing every 5 minutes. After centrifugation, the lysate’s supernatant was collected and combined with Streptavidin magnetic beads (50 µL, Thermo Scientific), incubated for 1 hour at 21°C, and then overnight at 4°C, to enrich biotinylated membrane proteins. The beads were washed, and biotinylated proteins were eluted sequentially with 100 µL of 50 mM biotin, 100 µL of elution buffer, and 100 µL of 5% SDS at 95°C.

The eluted proteins were reduced, alkylated, and processed for proteomics using S-Trap spin columns (ProtiFi) according to the manufacturer’s instructions (Matulis, 2016; HaileMariam et al., 2018). The resulting trypsin/LysC digested peptides were analyzed by LC-MS in data-dependent acquisition mode. Data analysis was performed using FragPipe v22.0 (Yu et al., 2021). Candidate receptor proteins for PvRBP1a_158–650_ were selected based on the presence of extracellular regions that are favorable for ligand interaction, evaluated using UniProt GO annotations and the TMHMM 2.0 predictor (Hallgren et al., 2022). Receptor candidates in the PvRBP1a_158-650_LTID sample that were detected in both duplicates and of significantly higher abundance (≥ 2 fold) compared to the negative control (LTID) were also considered. Subsequently, a parallel reaction monitoring (PRM) method was applied to validate and quantify the peptides of interest (as detailed in the **Supplementary Data**).

#### 2.6.5 Binding affinities of PvRBP1a_158–650_ to select receptor candidates by ELISA

The recombinant extracellular protein domains of receptor candidates TfR1 (Cys89-Phe760, SinoBiological), BSG (Met1-His205, SinoBiological), and full-length PHB2 (Origene) were used to evaluate the interaction between the PvRBP1a_158–650_ and its binding membrane proteins (**Supplementary Figure S1**). Maxisorp plates were coated in triplicates with 5 μg/mL of each protein for 2 hours at room temperature and blocked with SuperBlock Buffer (Thermo Scientific). Serial dilutions of PvRBP1a_158-650_LTID and LTID were prepared in blocking solution, ranging from 48,000 pM to 187.5 pM (1:2 dilution) and 4,000 pM to 1.28 pM (1:5 dilution), and incubated for 16 hours at 4°C. Bound PvRBP1a_158-650_LTID and LTID were detected with a TurboID-specific polyclonal rabbit antibody (anti-BirA mutated/TurboID, Agrisera, 1:10,000). After five washes with PBST, 100 µL of 3,3’,5,5’,-Tetramethylbenzidine (TMB) substrate was added, and the reaction was stopped with 50 µL of 1 M phosphoric acid. Absorbance was measured at 450 nm. Dissociation constants (Kd) were determined using GraphPad Prism v10.3.1 with non-linear regression and a one-site binding saturation model.

## 3 Results

### 3.1 *P. vivax* can invade the erythroid cell lines BEL-A and JK-1

**Figure 2:**
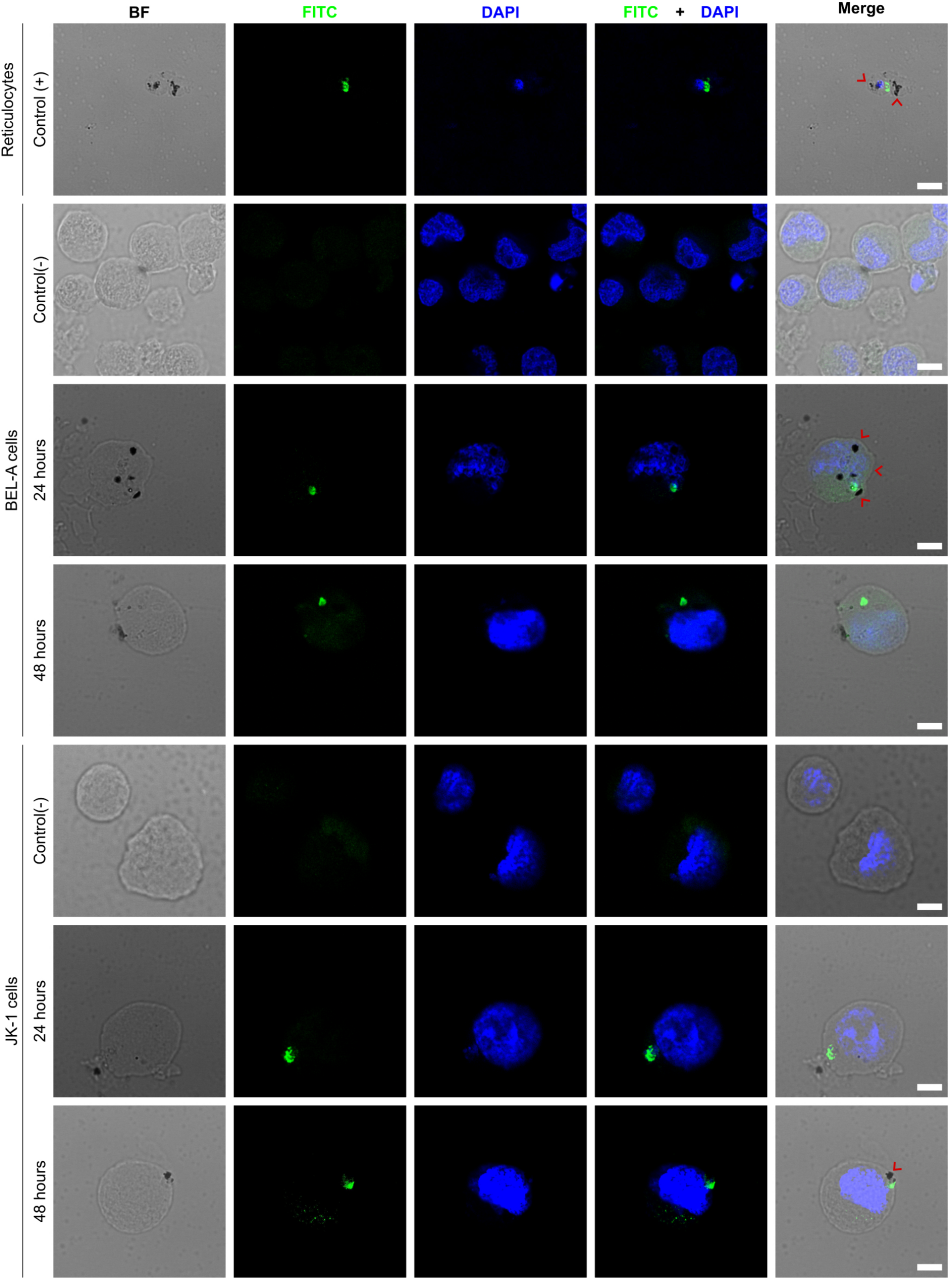
P*. vivax* invades BEL-A and JK-1 cells. The micrographs show *P. vivax*-infected reticulocytes as the positive Control (+); infected BEL-A and JK-1 cells at 24 and 48 h, and their non-infected negative Control (-); *P. vivax* lactate dehydrogenase (FITC green); DNA (DAPI blue); hemozoin crystals (black dots marked with red arrows); BF (bright field); Scale bars are 5 µm.

Cultured BEL-A and JK-1 cells were successfully invaded by *P. vivax* from a validated mono-infected malaria patient’s blood sample. The experiment required forgoing enrichment of the RBCs to 4.0% parasitemia (**Figures 1A–D**). Parasite invasion was confirmed by immunofluorescence microscopy, detecting the intracellular presence of *P. vivax* lactate dehydrogenase (PvLDH), which all blood stages of the parasite are known to express (Cao et al., 2024). PvLDH was detected in the positive control of infected reticulocytes, and in BEL-A and JK-1 cells, incubated with infected reticulocytes (**Figure 2**, FITC). The PvLDH signal was absent from non-infected control cells. Moreover, the presence of hemozoin (Hz) pigment, characteristic for hemoglobin consumption by Plasmodia within infected RBCs (Pandey and Tekwani, 1996), was observed (**Figure 2**, bright field, and merge). The dark Hz pigment was visible inside the parasite-infected nucleated erythroid cells as well as in infected reticulocytes that originated from the donor. Hz is an insoluble, crystallized digestion product of heme derived from the digestion of hemoglobin by malaria parasites, containing heme-derived β-hematin, which neutralizes the toxicity of free heme released after parasite invasion through a digestive process that involves the digestive vacuole structure (Coronado et al., 2014). On days 3 to 6 post-infection, no parasite-infected erythroid cells were observed, and cell mortality had substantially increased. Therefore, the experiment was stopped on day 6.

### 3.2 Overlapping membrane proteomes reveal potential *P. vivax* invasion receptors

Because *P. vivax* was able to invade the erythroid BEL-A and JK-1 cells, their membranes must contain the same essential receptor molecules as reticulocytes that enable parasite invasion. Furthermore, the membranes of mature erythrocytes are expected to lack these receptors or to express them only at insufficient abundances. Consequently, a quantitative comparison of the membrane proteome of these cells with those of human reticulocytes and erythrocytes identified potential receptors for *P. vivax* merozoite ligands that are most likely responsible for its reticulocyte-restricted invasion.

Stringent isolation procedures were necessary to obtain membrane proteins of pure reticulocytes. The isolated reticulocytes (CD71^+^, CD45^-^) used in this proteomic comparison had a purity of 98.4% (**Supplementary Figure S2**). Expression of CD71 is diminished during maturation into fully functional erythrocytes (Malleret et al., 2015b). Simultaneous determination of CD45 negativity was necessary, as CD45^+^ leukocytes also express CD71, to ensure purity of the isolated reticulocytes.

The BEL-A and JK-1 cells used in the membrane proteomic comparisons were harvested from *in vitro* cultures and exhibited distinct nucleated erythroid maturation stages, including proerythroblasts, basophilic erythroblasts, polychromatic erythroblasts, and orthochromatic erythroblasts (**Supplementary Figure S3**), with slight dominance of the basophilic and polychromatic stages.

In total, 2,100 proteins were identified in BEL-A cells and 2,178 in JK-1 cells. The number of proteins was lower in reticulocytes (1,234) and in erythrocytes (1,347). After filtering this data for membrane proteins (see **Supplementary Data**, **Supplementary Figure S4**), 1,530 and 1,595 such proteins were obtained from BEL-A and JK-1 cell ghosts, respectively, while 846 and 974 proteins were identified for reticulocyte and erythrocyte ghosts.

**Figure 3:**
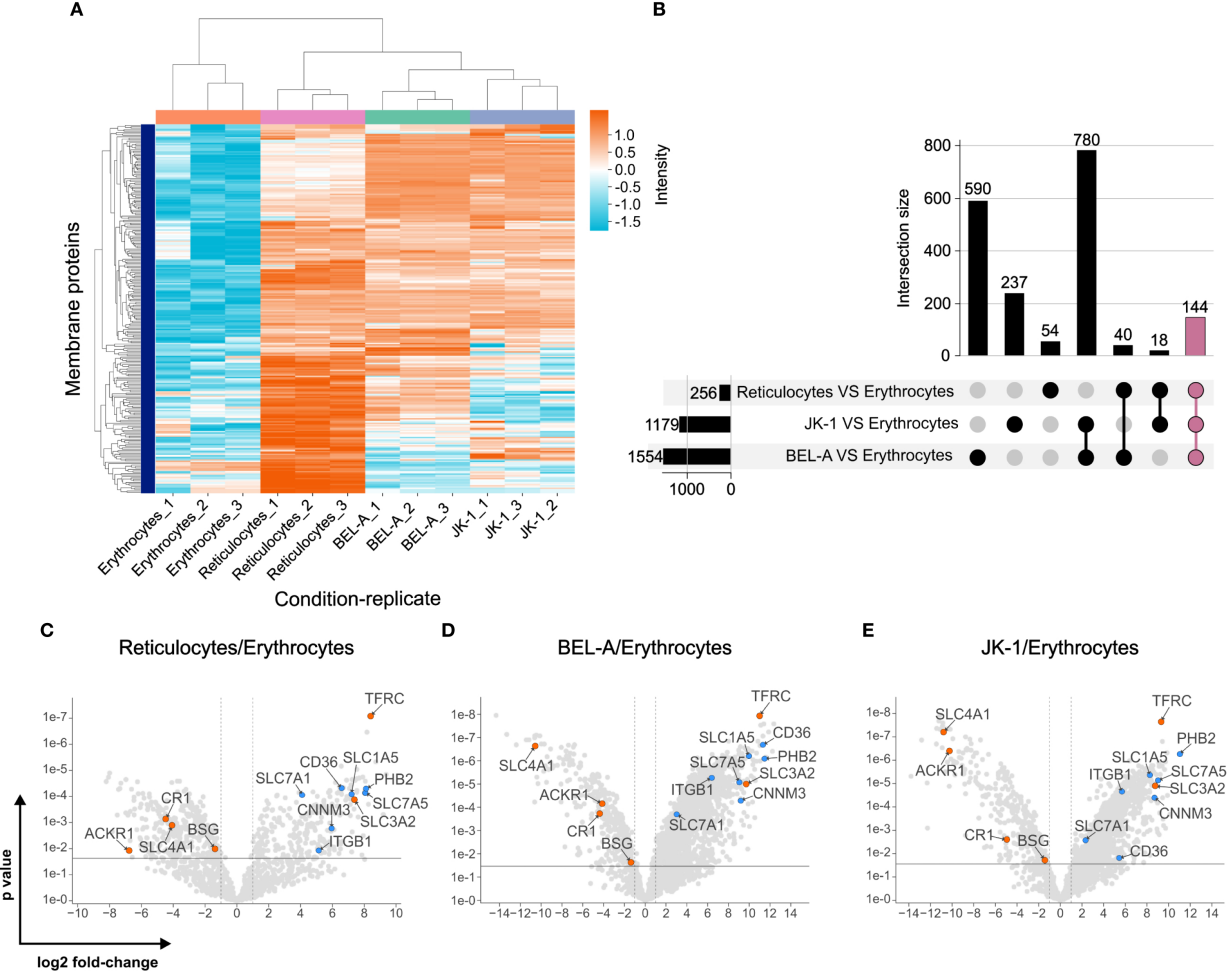
Plasma membrane proteomes of reticulocytes resemble those of erythroid cell lines. **(A)** Clustering of plasma membrane proteins in JK-1, BEL-A, reticulocytes, and erythrocytes, measured in triplicate. Log2 intensities. **(B)** Abundance of intersecting membrane proteins in reticulocytes, JK-1, and BEL-A cells compared to erythrocytes, represented in an UpSet plot (Lex et al., 2014). 144 proteins share higher abundance among cell lines and reticulocytes but are reduced in erythrocytes (mauve bar). **(C-E)** Putative receptors (blue) and characterized receptors (orange) for *P. vivax* merozoite invasion. Gene names are displayed instead of protein names for simplicity. The x-axis represents the log2 fold change, and the y-axis shows the P-value, indicating statistical significance.

Changes in membrane protein abundance were assessed by comparing erythroid cell lines and reticulocytes to mature erythrocytes. The protein abundancies of reticulocytes clustered better with those of BEL-A and JK-1 cells than with those of erythrocytes (**Figure 3A**). It was found that compared to erythrocytes 256 proteins were more abundant in reticulocytes, 1,179 in JK-1, and 1,554 in BEL-A. Of these, 144 identical proteins were increased in reticulocytes, JK-1, and BEL-A. However, reticulocytes and JK-1 cells share 18 proteins that are less abundant in erythrocytes, while BEL-A and reticulocytes have 40 proteins in common, that are less abundant in erythrocytes. Only 54 membrane proteins with higher abundance than in erythrocytes were identified exclusively in reticulocytes, 237 in JK-1, and 590 in BEL-A cells (**Figure 3B**).

When comparing the membrane protein abundance in reticulocytes, cell lines, and erythrocytes, known *P. vivax* receptors such as TfR1 (CD71), CD98hc, ACKR1/DARC, BSG, CR1, and band 3 (SLC4A1) were identified. TfR1 and CD98hc, which are lost during reticulocyte maturation to erythrocytes, were significantly more abundant in reticulocytes and cell lines. In contrast, the other receptors showed higher levels in erythrocytes (**Figures 3C–E**).

*In silico* topological analysis of membrane proteins enriched in reticulocytes and erythroid cell lines identified several candidates — CD98lc (SLC7A5), high-affinity cationic amino acid transporter 1 (CAT-1, SLC7A1), neutral amino acid transporter B0 (ATB(0), SLC1A5), CD36, Integrin β-1 (ITGB1), prohibitin-2 (PHB2), and the metal transporter CNNM3 — as possessing sizable extracellular regions that are potentially accessible for interaction with *P. vivax* merozoite ligands (**Supplementary Table S1**, **Supplementary Figure S5**).

The significantly higher abundance of these proteins in reticulocytes, BEL-A, and JK-1 cells compared to erythrocytes (**Figures 3C–E**; **Supplementary Table S1**) highlights them as potential candidates for *P. vivax* merozoite protein receptors, which may explain the parasite’s exclusivity for reticulocyte invasion.

### 3.3 Receptors for PvRBP1a_158-650_LTID identified via proximity labeling

**Figure 4:**
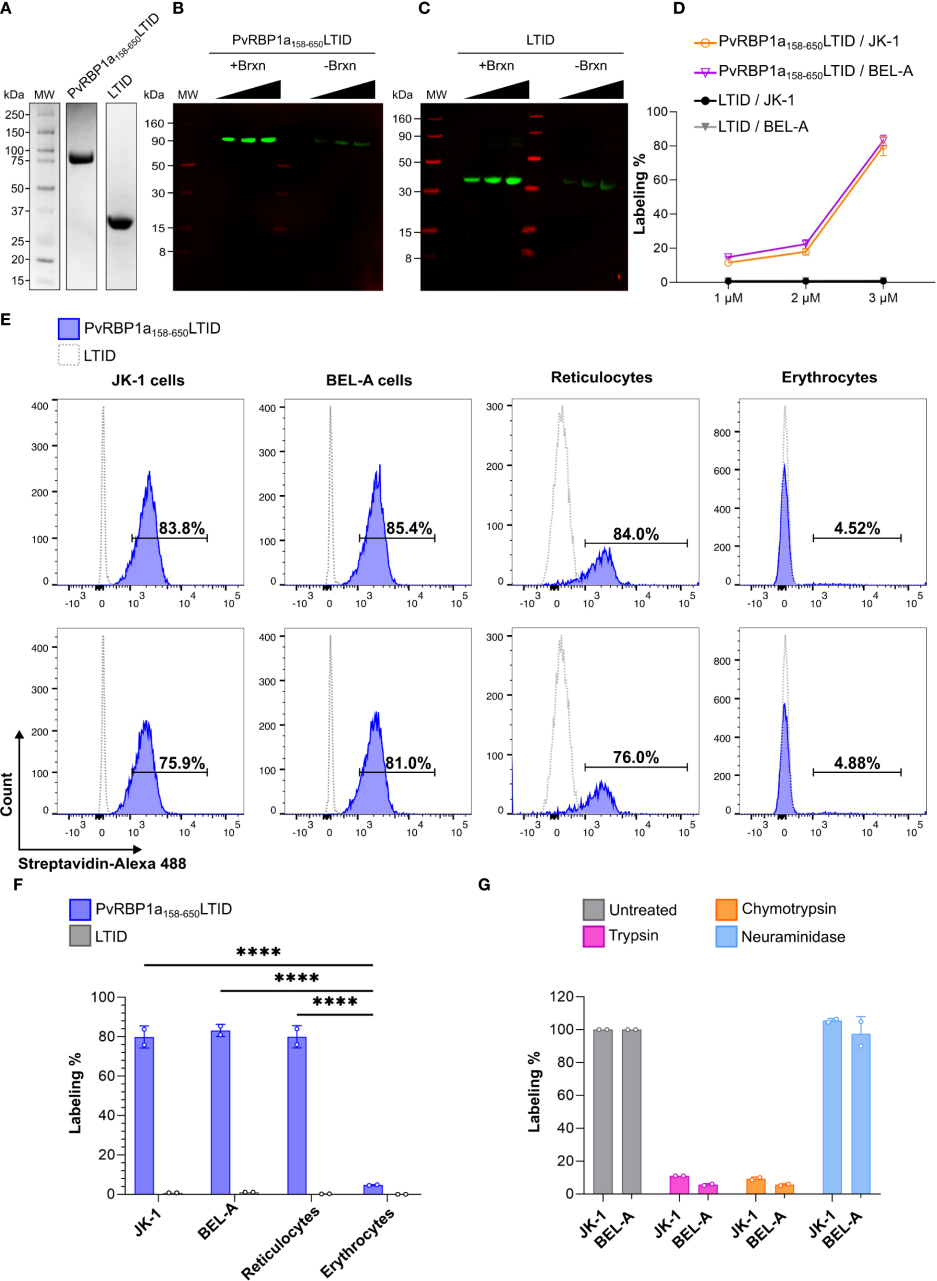
Proximity labeling with PvRBP1a_158-650_LTID in JK-1, BEL-A, reticulocytes, and erythrocytes. Recombinantly expressed soluble PvRBP1a_158-650_LTID and LTID by **(A)** SDS PAGE **(B, C)** auto-biotinylation assay with increasing concentrations of each protein at 1, 2, and 3 µM, detected with IRDye 800 Streptavidin (green bands), LICORbio molecular weight markers (MW, red bands); **(D)** biotin labeling of JK-1 (orange) and BEL-A (fuchsia) cells, in presence of PvRBP1a_158-650_LTID or LTID (negative control, JK-1 - black, and BEL-A - gray) at 1, 2, and 3 µM. **(E-G)** Flow cytometry **(E)** histograms of erythroid cells and human RBCs in presence of PvRBP1a_158-650_LTID (blue) or LTID (grey), both at 3 µM, demonstrating **(F)** significant degrees of cell surface labeling by PvRBP1a_158-650_LTID in erythroid cells and reticulocytes compared to erythrocytes (****p ≤ 0.0001). **(G)** Receptors to PvRBP1a_158–650_ are sensitive to cell surface treatment with trypsin and chymotrypsin but resistant to neuraminidase (labeling % as normalized to untreated cells).

To identify potential receptors of the PvRBP1a_158-650_LTID protein, the TurboID proximity labeling technique was used. This technique enables the biotinylation of proteins that come into close contact with the fused protein (within 10 nm), facilitating the identification of their interactions (Branon et al., 2018; Cho et al., 2020). Therefore, the fusion protein PvRBP1a_158-650_LTID and the LTID control were obtained in soluble form, with PvRBP1a_158-650_LTID having a molecular weight of ~94 kDa and LTID ~36 kDa (**Figure 4A**). Both proteins exhibited enzymatic activity and self-biotinylation at 1, 2, and 3 µM (**Figures 4B, C**).

### 3.4 PvRBP1a_158-650_LTID exhibits comparable binding to reticulocytes and erythroid cell lines via a proteinaceous receptor

Proximity biotinylation mediated by TurboID facilitated binding evaluation through the biotin-streptavidin interaction. The assays showed that PvRBP1a_158-650_LTID binding is concentration-dependent (**Figure 4D**). Since the highest percentage of biotin labeling on the cell surface was obtained at 3 µM, this concentration was selected to analyze PvRBP1a_158-650_LTID binding to enriched reticulocytes (87.5% purity) (**Supplementary Figure S6**), erythrocytes, and cell lines. The results showed that PvRBP1a_158–650_ had 80% biotinylation on the surface of reticulocytes, 4.7% on erythrocytes, and 79.85% and 83.2% on the surface of JK-1 and BEL-A cells, respectively. A statistically significant difference was found between the cell lines and reticulocytes compared to erythrocytes (P ≤ 0.0001). However, no significant difference was observed between the cell lines and reticulocytes (**Figures 4E, F**). These data suggest that erythroid cell lines exhibit PvRBP1a_158–650_ binding activity comparable to reticulocytes, indicating that they may express the receptor for this specific *P. vivax* ligand on their surface. Additionally, no labelling was detected with LTID, confirming the specificity of the PvRBP1a_158–650_ receptor interaction.

Cell surface labelling with PvRBP1a_158-650_LTID was sensitive to trypsin and chymotrypsin treatment but resistant to neuraminidase, which removes sialic acid from glycans that modify proteins in vertebrates (**Figure 4G**). Therefore, the cell surface receptor function for PvRBP1a_158–650_ is proteinaceous and not dependent on sialic acid-terminated glycans.

### 3.5 Enrichment of PvRBP1a_158-650_LTID biotinylated cell surface proteins, identifies TfR1 and prohibitin-2 as the likely reticulocyte-restricting receptors

**Figure 5:**
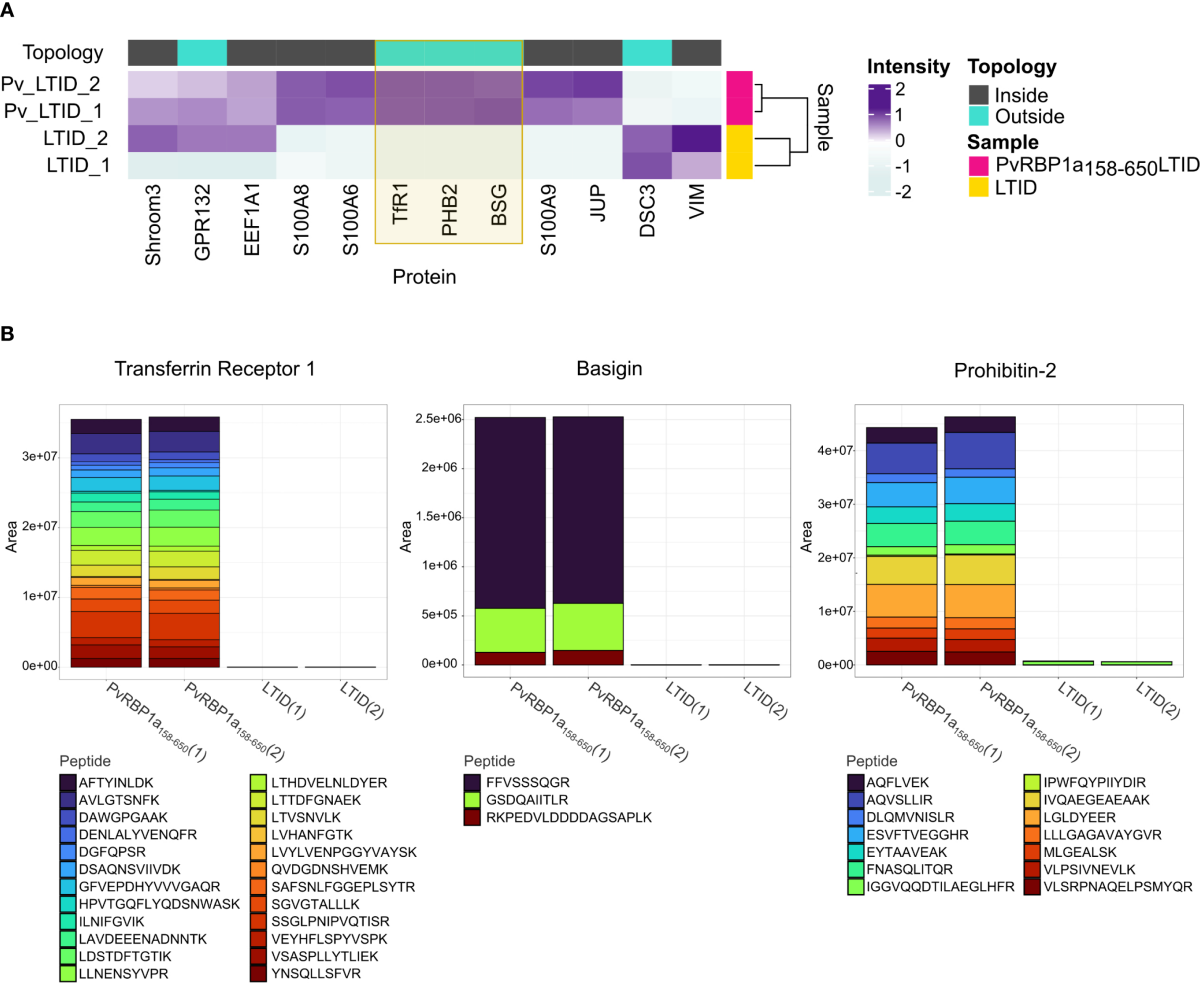
Proximity biotin-labeled TfR1 (CD71), Basigin, and Prohibitin-2, are identified as *P. vivax* receptor candidates after interaction of JK-1 cells with PvRBP1a_158-650_LTID and validated by targeted LC-MS. **(A)** Heatmap of labeled plasma membrane protein intensities in the PvRBP1a_158-650_LITD (fuchsia) and LTID (yellow) control samples, analyzed in duplicate by DDA LC-MS proteomics. Intensities are represented on a Z-score scale, where each value was transformed by the number of standard deviations (SD) from the mean. Topology categorizes proteins by their cellular localization: intracellular (Inside, dark gray) and those with extracellular domains (Outside, cyan). They include Shroom3, GPR132 – probable G-protein coupled receptor 132, EEF1A1 – Elongation factor 1-alpha 1, protein S100-A8, protein S100-A6, TfR1, PHB2 – prohibitin-2, BSG, protein S100-A9, JUP – junction plakoglobin, DSC3 – Desmocollin-3, and VIM – vimentin. **(B)** Validated interactions of transferrin receptor 1, basigin, and prohibitin-2 with PvRBP1a_158-650_LITD, but not with LITD, during the TurboID procedure, followed by PRM LC-MS. (1) (2) – duplicates. Stacked bars are the sum of the LC-MS ion chromatographic peak areas of the trypsin digested peptides (colored boxes) of each protein, indicating the contribution of each peptide to the individual protein abundance.

PvRBP1a_158-650_LTID biotinylated membrane proteins from erythroid cells were captured via streptavidin-affinity and subjected to proteomics analysis, revealing a total of 278 proteins, of which 12 were localized to the plasma membrane. However, the LTID control contained five of these proteins, leaving seven unique to enrichment after biotinylation with PvRBP1a_158-650_LTID. Four of these membrane proteins do not possess extracellular regions, while TfR1, prohibitin-2, and BSG do. Such extracellular regions should be required to facilitate an interaction with *P. vivax* merozoite ligands (**Figure 5A**). Interaction of PvRBP1a_158–650_ was successfully validated by targeted PRM LC-MS analysis for TfR1, BSG, and prohibitin-2 (**Figure 5B**). It clearly demonstrated that only PvRBP1a_158-650_LTID biotinylated these three proteins, while LTID did not.

BSG is more abundant in erythrocytes than in reticulocytes and in the erythroid cell lines. In contrast, TfR1 and prohibitin-2 were significantly less abundant in erythrocytes (**Figures 3C–E**). In fact, TfR1 and prohibitin-2 were among the most abundant membrane proteins in reticulocytes and in the erythroid cell lines JK-1 and BEL-A. These findings suggest that PvRBP1a_158–650_ likely facilitate the recognition and invasion of reticulocytes through interaction with TfR1 and prohibitin-2.

### 3.6 PvRBP1a_158-650_LTID interacts with high-affinity binding to TfR1, BSG, and prohibitin-2

**Figure 6:**
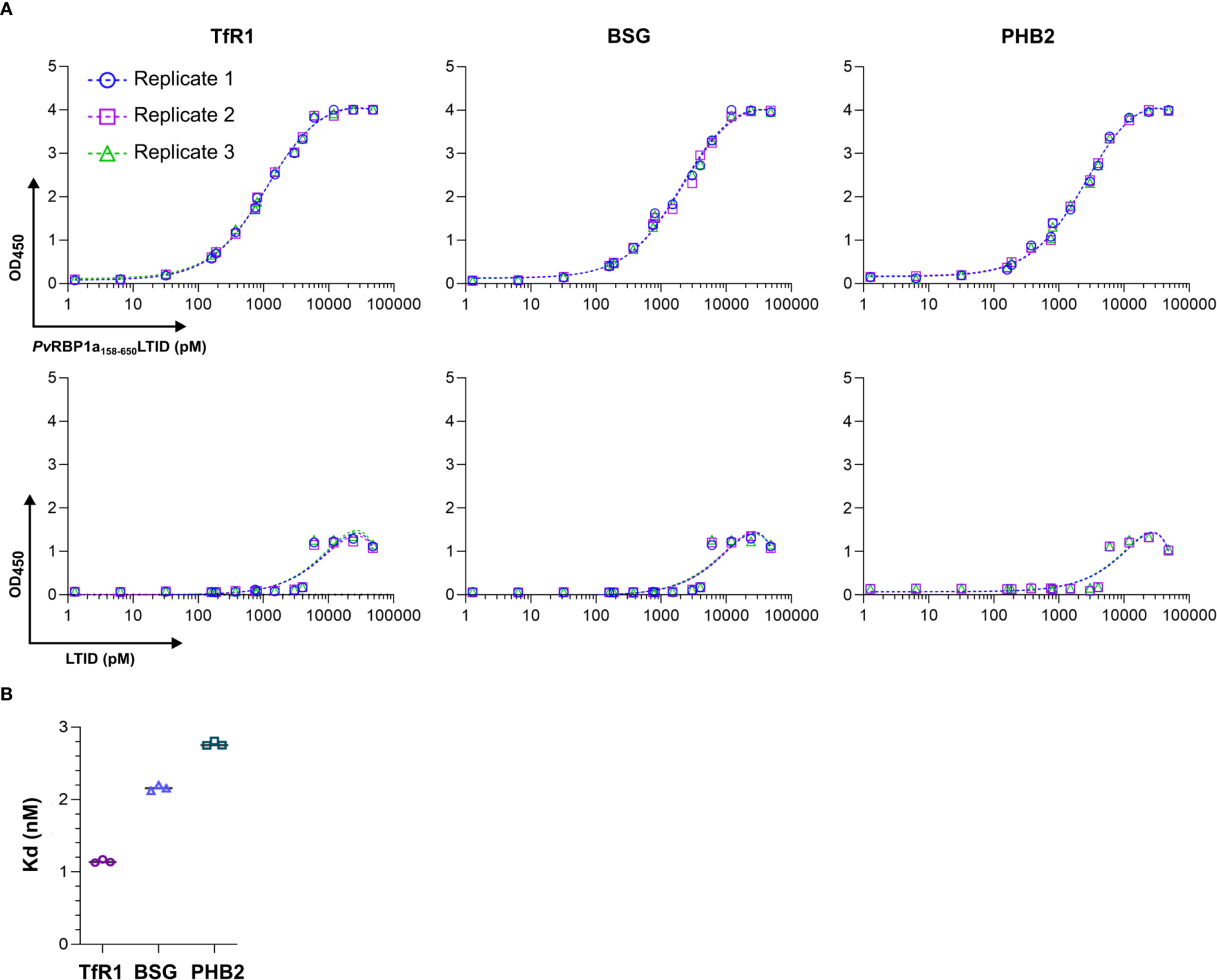
PvRBP1a_158–650_ binds to TfR1, BSG, and PHB2 at nanomolar affinities. **(A)** Titration ELISAs of protein-protein interactions between soluble ligand PvRBP1a_158-650_LTID, control LTID, and the immobilized receptor candidates TfR1, BSG, and PHB2. OD450 absorbance values represent the binding of a TurboID-specific HRP-labeled antibody for the biotin-free quantification of ligand and control in triplicate, fitted by a single-site binding model; **(B)** average dissociation constants (kd) summarized as determined from the fitted titration curves above.

The titration curves fit well to a single-site binding saturation model. In contrast, the negative control LTID displayed a nonspecific binding pattern “unstable”, corroborating the specificity of the interactions (**Figure 6A**). The Kd obtained from the ELISA titration indicated that PvRBP1a_158-650_LTID had high affinity for TfR1 (Kd: 1.15 nM), followed by BSG (Kd: 2.16 nM) and prohibitin-2 (Kd: 2.77 nM) (**Figure 6B**).

## 4 Discussion

This study demonstrates the utility of erythroid cell lines JK-1 and BEL-A as suitable surrogates for reticulocytes for studying the invasion process of the *P. vivax* malaria parasite. While these cell lines and their culture conditions did not support a continuous *P. vivax* culture *in vitro*, the formation of the Hz pigment and immuno detection of PvLDH strongly supported parasite invasion. In contrast to human reticulocytes, both erythroid cell lines are nucleated, which might permit them to initiate a cell-death program upon parasite invasion. Consistently, JK-1 cells were previously reported to support cell entry by both *P. vivax* and *P. falciparum* (Kanjee et al., 2017; Gruszczyk et al., 2018b), while BEL-A cells have so far only been studied with *P. falciparum* (Satchwell et al., 2019). This study is the first to report *P. vivax* invasion of BEL-A cells, confirming their susceptibility alongside JK-1 cells.

The quantitative membrane proteome comparison of reticulocytes and erythroid cell lines with erythrocytes revealed Prohibitin-2 (PHB2), TfR1 (CD71), the CD98 heavy chain (4F2hc, gene SLC3A2), the CD98 light chain (LAT-1, gene SLC7A5), ATB(0) (gene SLC1A5), CAT-1 (SLC7A1), CD36, Integrin β-1 (gene ITGB1), and Metal transporter CNNM3 to be of significantly increased abundance in those cell lines and reticulocytes. Whereas they are strongly decreased (practically absent) in fully matured erythrocytes. The increased abundance of TfR1 and CD98 in reticulocytes over erythrocytes is consistent with previous studies (Malleret et al., 2015b, 2021). However, genetic manipulation (Gruszczyk et al., 2018a) or antibody blockade (Malleret et al., 2021) of these proteins only partially reduced *P. vivax* invasion, suggesting the involvement of additional receptors. The heavy chain of CD98 (SLC3A2) was reported to be bound by *P. vivax* in immature RBCs via PvRBP2a (Malleret et al., 2021). Therefore, other potential *P. vivax* receptor candidates with extracellular regions, namely Prohibitin-2, the CD98 light chain (LAT-1), ATB(0), CAT-1, CD36, Integrin β-1, and CNNM3 should be considered. These membrane proteins participate in various protein-protein interactions that facilitate the entry of microorganisms into host cells (Albritton et al., 1993; Yoshimoto et al., 1993; Smith et al., 1998; Tailor et al., 1999; Graham et al., 2003; Weigel-Kelley et al., 2003; Maginnis et al., 2006; Xiao et al., 2008; Feire et al., 2010; Nägele et al., 2011; Wintachai et al., 2012; Su et al., 2020; Olaya-Galán et al., 2021). Interestingly, LAT-1, that together with its heavy chain 4F2hc forms the heteromeric CD98 (Lee et al., 2019; Yan et al., 2019), plays a role in hepatitis C virus entry (Nguyen et al., 2018), raising the question of whether *P. vivax* may also interact with LAT-1.

Proximity labeling of erythroid cells with PvRBP1a_158-650_LTID was largely consistent with previous observations, in which ~50% of reticulocytes binding and ~20% of erythrocytes bound to PvRBP1a_157-650_ (Ntumngia et al., 2018). Furthermore, 20 of reticulocytes and only 1% of erythrocytes bound to PvRBP1a351-599 (Han et al., 2016), while 31.5% of reticulocytes were reported to bind to PvRBP1a30-778 (Gupta et al., 2017). Additionally, the trypsin and chymotrypsin sensitivity of these recombinant protein (Han et al., 2016; Gupta et al., 2017; Ntumngia et al., 2018), as well as the PvRBP1a157–653 HAPBs (Urquiza et al., 2002) align with our results.

PvRBP1a_158–650_ was found to interact with Prohibitin-2, TfR1, and BSG. Prohibitin-2 and TfR1 are more abundant in reticulocyte membranes and cell lines compared to erythrocytes, while BSG is more abundant in erythrocytes. These findings suggest that PvRBP1a_158–650_ may facilitate reticulocyte recognition and invasion through interaction with Prohibitin-2 and TfR1. Additionally, interaction with BSG may contribute to binding activity to erythrocytes, but not their restricted invasion, consistent with previous studies on PvRBP1a binding (Urquiza et al., 2002; Han et al., 2016; Gupta et al., 2017; Ntumngia et al., 2018).

This study demonstrated strong binding of PvRBP1a_158–650_ with the 89–760 domain of TfR1, contrasting with prior work that did not detect this interaction, possibly due to the crucial role of TfR1’s 89–120 region, not included in the previous protein construct (Gruszczyk et al., 2018b). TfR1 is a known receptor for PvRBP2b (Gruszczyk et al., 2018a), as well as for various New World arenaviruses (Radoshitzky et al., 2007, 2008).

Prohibitin-2 has been implicated in facilitating the entry of diverse viruses, including enteroviruses, coronaviruses, HIV-1, and flaviviruses such as dengue (Cornillez-Ty et al., 2009; Emerson et al., 2010; Kuadkitkan et al., 2010; Su et al., 2020). Although traditionally characterized as a protein of the inner mitochondrial membrane and nucleus, subsequent studies have demonstrated its presence at the plasma membrane, notably in CHME-5 microglial cells and RMS cells (Wintachai et al., 2012; Fu et al., 2013). Its established function as a receptor or co-receptor for several pathogens further supports the notion that prohibitin-2 can localize to the cell surface, where it may contribute to pathogen attachment and entry.

BSG, a known receptor for *P. falciparum* RH5 (Crosnier et al., 2011; Chen et al., 2014), a member of the PfRH family homologous to the PvRBP proteins of *P. vivax* (Rayner et al., 2000; Triglia et al., 2001), also serves as a receptor for *P. vivax* TRAg38 (Rathore et al., 2017). In addition, PvRBP1 is the orthologue of *P. falciparum* normocyte binding protein 1 (PfNBP1) (Rayner et al., 2001), and PvRBP1a (N352–K598) shares sequence homology with PfRH4 (N328–D588) (Gaur et al., 2007), although these proteins engage different host receptors. Interestingly, PvRBP2a, which binds TfR1 in *P. vivax*, displays a structural scaffold similar to PfRH5 of *P. falciparum* (Gruszczyk et al., 2016), highlighting that orthologous and homologous relationships can provide an evolutionary framework to interpret invasion mechanisms even when receptor usage differs. Such cross-species comparisons are a common approach in malaria research (Tebben et al., 2022) and contextualize our findings on PvRBP1a interactions.

This research demonstrated the binding versatile of PvRBP1a_158-650_, which interacts with three different membrane proteins. In biological systems, ligands often bind multiple receptors, as seen with *Plasmodium* interactions; PvTRAg38 binds both BSG and band 3 (Alam et al., 2016, p. 3; Rathore et al., 2017), and PfEMP1binds to several receptors (Yipp et al., 2000; Vogt et al., 2003; Vigan-Womas et al., 2012; Esser et al., 2014; Kessler et al., 2017).

This study demonstrates that the BEL-A and JK-1 cells are suitable models for studying *P. vivax* receptor-ligand interactions, providing viable alternatives to reticulocytes. The similarity in the abundance of potential receptor candidates between cell lines and reticulocytes, and their dissimilarity with erythrocytes, validates the use of JK-1 and BEL-A cell lines as surrogate models for the study of *P. vivax* merozoite ligand-receptor interactions, and suggests the existence of other potential *P. vivax* receptors. prohibitin-2 and TfR1 may contribute to a redundant reticulocyte-restricted invasion pathway because they exhibit high binding affinities to PvRBP1a_158-650_. These findings lay the foundation for the comprehensive study of all *P. vivax* invasion mechanisms and for the development of targeted therapies against malaria.

## Data Availability

LC-MS DIA data was deposited to the MassIVE repository at the Center for Computational Mass Spectrometry, University of California, San Diego under Dataset Identifier: MSV000093438 (https://doi.org/doi:10.25345/C5W669K65). The DDA and PRM LC-MS data sets are available under MSV000096045 (https://doi.org/doi:10.25345/C5Q52FR0R).
