# Supplementary material for: The reticulocyte restriction: invasion ligand RBP1a of Plasmodium vivax targets human TfR1, prohibitin-2, and basigin
Source: Front Cell Infect Microbiol. 2025 Sep 25;15:1671048. doi: 10.3389/fcimb.2025.1671048 (PMC12507909; doi:10.3389/fcimb.2025.1671048)
Supplement: Supplementary file 2 [file DataSheet2.pdf]

Supplementary Material

1 Supplementary Data

1.1 Detection of *Plasmodium* species by nested PCR

1st amplification: Each PCR reaction (25 µL total volume) contained primers rPLU 6 (TTAAAATTGTTGCAGTTAAACG) and rPLU 5 (CCTGTTGTTGCCTTAAACTTC) at 1µM each (Snounou et al., 1993), - 5 µL of DNA template (100 ng), and GoTaq Green Master Mix (Promega, 1X). The conditions for the first amplification step were: 95°C for 5 minutes (1X); 94°C for 1 minute, 58°C for 2 minutes, 72°C for 2 minutes (30X); followed by 72°C for 5 minutes (1X).

2nd amplification: Subsequently, the 1:35 diluted primary PCR product was used as the template in the secondary PCR, which was conducted with the species-specific primers: rFAL 1 (TTAAACTGGTTTGGGAAAACCAAATATATT) and rFAL 2 (ACACAATGAACTCAATCATGACTACCCGTC) for P. *falciparum*; and rVIV1 (CGCTTCTAGCTTAATCCACATAACTGATAC) and rVIV2 (ACTTCCAAGCCGAAGCAAAGAAAGTCCTTA) for *P. vivax*, each at 1µM (Snounou et al., 1993).

The PCR temperature profile was 94°C for 5 minutes (1X); 94°C for 30 seconds, 60°C for 1 minute, 72°C for 1 minute (35X); followed by 72°C for 5 minutes (1X). Genus-specific amplicons were separated by electrophoresis on a 2% agarose gel stained with SYBR Safe. The *P. vivax* amplicon was sequenced by Sanger and found to be identical with the *P. vivax* small subunit 18S ribosomal RNA (GenBank XR_003001206.1).

Positive control templates for *P. falciparum* and *P. vivax*, and the negative control, healthy human genomic DNA, were from our validated collection.

1.2 Immunofluorescence assay (IFA)

BEL-A and JK-1 cells infected with *P. vivax* were fixed on immunofluorescence microscope slides with 4% paraformaldehyde in PBS. After washing and permeabilizing with 1% Triton X-100, cells were blocked with SuperBlock Buffer (Thermo Scientific) for 30 minutes. They were then incubated overnight at 4°C with mouse anti-PvLDH antibody (1:100, The Native Antigen Company) in SuperBlock Buffer diluted 10-fold and supplemented with 0.05% Tween-20. After five washes with PBS, the slides were incubated with goat anti-mouse IgG –FITC antibody (1:200, Sigma-Aldrich) for 1 hour, washed again, and stained with 300 nM DAPI for 5 minutes. Cells were mounted in VECTASHIELD media, sealed, and imaged with a Zeiss LSM 880 confocal microscope. Images were processed utilizing ImageJ 1.53s (Schneider et al., 2012).

1.3 Collection of blood samples from non-infected humans for proteomics analysis

500 mL of total peripheral blood were obtained from three patients undergoing therapeutic phlebotomy for high iron content. To separate the RBCs from the plasma and buffy coat, the blood was centrifuged at 1,900 × g for 5 minutes at 4°C, and the RBCs were washed three times in PBS. To limit proteolysis, the samples were kept at 4°C throughout every step.

To determine the percentage of reticulocytes, washed RBCs were diluted 1:10 with PBS and incubated with APC anti-Human CD71 (BD Pharmingen) and APC-Cy7 anti-Human CD45 (BD Pharmingen) antibodies (1:10 each) for 30 minutes at 4°C. The $CD71^+$ $CD45^-$ population was analyzed by flow cytometry using a FACSAria Fusion instrument (BD).

Reticulocytes were enriched by Percoll density gradient centrifugation, layering 6 mL of washed RBCs onto 6 mL of Percoll (3 mL 70% and 3 mL 62%). After centrifugation at 3,880 × g for 30 minutes at 4°C with brakes off, the reticulocyte-enriched fraction was collected, washed, and stained with anti-CD71 and anti-CD45 antibodies as described. The sample was then centrifugated at 150 × g, resuspended, and sorted on a FACS Aria Fusion (BD) to collect the $CD71^+$ $CD45^-$ population. Percoll-enriched and sorted samples were further evaluated by flow cytometry. The resulting purified reticulocytes were used to generate ghost reticulocytes (gRet).

For additional verification, both the initial and Percoll-enriched samples were stained with brilliant cresyl blue and analyzed under Bright-field microscopy at 63X.

Erythrocytes, depleted of reticulocytes, were collected after Percoll density gradient centrifugation. 5 mL of erythrocytes were washed with PBS, centrifuging at 1,900 × g for 5 minutes each time. Ghost erythrocytes (gEry) were subsequently prepared.

1.4 JK-1 and BEL-A collection and morphological characterization

JK-1 cells were cultured in IMDM GlutaMAX with 10% fetal bovine serum and 1X Penicillin-Streptomycin, while BEL-A cells were maintained in StemSpan SFEM medium with 50 ng/mL SCF, 3 U/mL EPO, 1µM dexamethasone, and 1µg/mL doxycycline. Both cell lines were cultured at 37°C with 5% CO2 at a cellular density of 70,000 – 150,000 cells/mL (Trakarnsanga et al., 2017). 1x107 cells from each culture were harvested, washed with PBS, and used to prepare ghost cells (gJK-1 and gBEL-A).

For morphological characterization, 1x105 cells of each line were seeded, fixed with methanol, and stained with Wright's eosin-methylene blue. Slides were analyzed by bright-field microscopy.

1.5 Ghost cell preparation

Reticulocytes, erythrocytes JK-1 and BEL-A cells were washed ~10 times and centrifuged at 17,000 × g for 15 minutes at 4°C with ghost lysis buffer (5 mM NaH_2PO_4, 10 mM NaCl, 0.5 mM EDTA and 1 mM PMSF, pH 8,0,) until white pellets of ghosts were obtained.

1.6 Ghost cell protein extraction / membrane protein extraction

gRet, gEry, gJK-1, and gBEL-A were incubated (1:1) with the protein extraction buffer (50 mM tetraethylammonium bromide (TEAB), 1X Halt protease inhibitor cocktail, 0.5 mM PMSF, 1% Triton X100 and 5% SDS) for 5 minutes at 95°C and centrifugated at 17,000 × g for 1 hour. Soluble proteins were recovered from the supernatants.

1.7 Quantitative comparison of membrane proteomes by Data Independent Acquisition (DIA) LC-MS/MS

Tryptic peptides were analyzed using an Exploris 480 orbitrap mass spectrometer with a 1200 Easy nanoLC system, using an Acclaim PepMap 100 pre-column (75µm × 2cm, nano Viper 2Pk C18, 3µm, 100 Å) and a PepMap RSLC C18 analytical reversed-phase column (75 µm × 25 cm, 2 µm, 100 Å Thermo, San Jose, CA, USA). Peptides were separated at a flowrate of 300 nL/min over a 195-minute gradient as follows: Buffer A consisted of 0.1% formic acid in water, and Buffer B was 80% acetonitrile with 0.1% formic acid. Gradient: 0 min at 2% B, 170 min to 33% B, 180 min to 100% B, and 195 min at 100% B. The full MS scan was acquired with an orbitrap resolution of 120K, and a range of 390-1100 m/z and an automatic gain control (AGC) target set to "standard". Fragment ions were generated by high collision dissociation with 27% normalized collision energy and acquired by data-independently (DIA-MS/MS). The extraction window sizes were set to a width of m/z 8 and ranged from m/z 400 to m/z 1000. A second set of extraction windows was shifted by m/z 4 to achieve a staggered acquisition pattern as described by Pino et. al (Pino et al., 2020a). DIA spectra were acquired with a normalized AGC target of 1000% and a maximum ion injection time of 60 ms at 30K resolution.

1.8 DIA LC-MS/MS Data Analysis

Spectra in raw files were centroided, demultiplexed and files were converted to the mzML format using MSConvert 3.0.21101 (Chambers et al., 2012). Protein identification and quantitation was done with DIA-NN 1.8.2 (Demichev et al., 2020), using the human UniProt reference proteome. Briefly, key parameters were: Enzymatic digestion with Trypsin, maximum of 2 missed cleavages, variable modifications for methionine oxidation and N-terminal acetylation, and fixed cysteine carbamidomethylation. The DIA-NN output files were processed and analyzed with R (R Core Team, 2023) and Mass Dynamics (Quaglieri et al., 2022).

1.9 Cloning, expression and purification of recombinant proteins

Recombinant plasmids pET-28a(+)PvRBP1a$_{158\text{-}650}$LTID and pET-28a(+)LTID were obtained through gene synthesis services by Twist Bioscience (San Francisco, CA, USA) and transformed into *E. coli* BL21 competent cells (New England Biolabs). Each transformation involved incubating 100 ng of plasmid with cells on ice for 30 minutes, followed by heat shock at 42°C for 10 seconds and a final 5-minute incubation on ice. Cells were then recovered in 950 µL SOC medium, incubated at 37°C for 1 hour, and plated on Luria-Bertani (LB) agar containing kanamycin for selection. Plates were incubated for 16 hours at 37°C to confirm successful transformation.

Cells transformed with pET-28a(+)PvRBP1a$_{158\text{-}650}$LTID were cultured in 50 mL LB medium supplemented with 50 µg/mL kanamycin at 37°C and 250 rpm for 16 hours. The culture was diluted 1:10 (v/v) into 500 mL Terrific Broth (TB) with 50 µg/mL kanamycin and incubated under the same conditions. Upon reaching an OD600 of 0.7–0.8, the culture was cooled to 4°C for 30 minutes, and expression was induced with 0.5 mM isopropyl β-D-1-thiogalactopyranoside (IPTG) at 26°C and 220 rpm for 16 hours. Cells were harvested by centrifugation at 2,400 ×g for 20 minutes. For pET-28a(+)LTID-transformed cells, growth and expression conditions were identical except for IPTG induction at 1 mM, performed at 30°C for 4 hours. Harvesting was conducted as described above.

The expressed proteins were purified using affinity chromatography. The supernatants obtained from cell lysates were incubated for 16 hours at 4°C with 5 mL of nickel-NTA agarose resin (Thermo Scientific), pre-equilibrated with phosphate buffer at pH 8.0.

To minimize nonspecific binding and remove weakly bound proteins from the resin, the PvRBP1a$_{158-650}$LTID protein mixture was washed with 10 column volumes of phosphate buffer (pH 8.0) containing 10 mM imidazole and 0.1% Triton X-100, followed by an additional wash with 100 mL of the same buffer without detergent. Elution was performed using 10 mL of phosphate buffer (pH 8.0) with increasing concentrations of imidazole (40 mM, 250 mM, and 500 mM). Similarly, the LTID protein mixture underwent the same washing procedure, and elution was performed using phosphate buffer with increasing concentrations of imidazole (50 mM, 100 mM, 250 mM, and 500 mM).

Finally, PvRBP1a$_{158-650}$LTID and LTID proteins were buffer-exchanged to phosphate buffer at pH 7.5 using Amicon Ultra centrifugal filters with molecular weight cutoffs of 50 kDa and 30 kDa (Millipore), respectively. Protein quantification was conducted by densitometry, and the purified proteins were stored at -80°C.

1.10 Reticulocyte purification for proximity labeling assays

Peripheral blood (20 mL) was collected into EDTA tubes, and RBCs were separated from plasma and buffy coat by centrifugation at 1,900 × g for 5 minutes at 4°C. To determine the reticulocyte percentage, washed RBCs were diluted 1:10 in PBS and stained with anti-human CD98-APC (1:10, Miltenyi Biotec) and anti-human CD45-APC-Cy7 (1:10, BD Pharmingen) at 4°C in the dark for 30 minutes. CD98^{+} CD45^{-} cells were analyzed using a FACSAria Fusion (BD).

Reticulocytes were enriched by layering 6 mL of washed RBCs onto a Percoll gradient (3 mL of 70% and 3 mL of 62%) and centrifuging at 3,880 × g for 30 minutes at 4°C without applying the brake during deceleration to preserve cell integrity. The enriched reticulocyte fraction was collected, washed twice with PBS, and stained again with the same antibodies. After centrifugation at 150 × g for 5 minutes, the pellet was washed twice with PBS. CD98^{+} CD45^{-} reticulocytes were sorted using a FACSAria Fusion (BD).

1.11 DDA LC-MS/MS Analysis of Proximity-labeled Biotinylated Membrane Proteins

Digested peptides were analyzed using an Orbitrap Exploris 480 mass spectrometer (Thermo Fisher) equipped with an Easy nanoLC 1200 HPLC system. For separation, a pre-analytical Acclaim PepMap 100 column (75 µm × 2 cm, nano Viper C18, 3 µm, 100 Å) and an analytical PepMap RSLC C18 reversed-phase column (75 µm × 25 cm, 2 µm, 100 Å, Thermo, San Jose, CA, USA) were used. Peptides were separated at a flow rate of 300 nL/min over a 75-minute gradient. Buffer A consisted of 0.1% formic acid in water, while Buffer B was 80% acetonitrile with 0.1% formic acid. The elution gradient was as follows: 0 min at 2% Buffer B, 5 min at 2% Buffer B, 60 min at 38% Buffer B, 65 min at 100% Buffer B, and 75 min at 100% Buffer B. Spectra were acquired using a data-dependent acquisition (DDA) method, with full MS scans performed at a resolution of 60,000 in the range of 400–1,800 m/z. Ions with a charge state between 2 and 8 and a minimum intensity of 4E4 were selected for fragmentation, with an isolation window of 1 m/z. Fragmentation was carried out with a normalized collision energy of 28%, and the resulting spectra were recorded at a resolution of 30,000.

Data analysis was performed using FragPipe v22.0 (Yu et al., 2021), applying the default parameters for closed searches. The human proteome with isoforms from UniProt was used, and protein quantification was conducted using IonQuant. To enrich membrane proteins, the plasma membrane localization ontology from UniProt was utilized.

1.12 Parallel Reaction Monitoring (PRM) Method

To validate protein quantification results from DDA, samples were re-analyzed using PRM mass spectrometry. This targeted approach enhances the accuracy and specificity of protein quantification by selecting predefined ions for fragmentation, improving confidence in detecting and quantifying peptides of interest. The same column and gradient as in the DDA method were used to maintain consistency.

PRM analysis was performed using an inclusion list of unique peptides from candidate receptor proteins for PvRBP1a$_{158\text{-}650}$, including retention time, m/z ratio, and charge state from DDA experiments. Fragmentation was conducted within a 10-minute window around the average retention time from the DDA. MS/MS scans were acquired at a resolution of 30,000. Data analysis was performed with Skyline (64-bit) 24.1 (Pino et al., 2020b), and results were summarized and visualized using R (R Core Team, 2023).

2. Supplementary Figures and Tables

2.1 Supplementary Figures

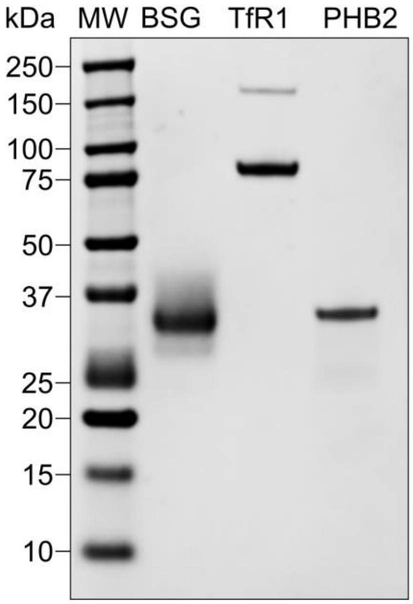

Supplementary Figure 1. Purity Analysis of Recombinant Proteins. Recombinant proteins BSG, TfR1, and PHB2 were separated on a 4–12% polyacrylamide gel and stained with Coomassie blue. The observed bands correspond to the expected molecular weights for each protein: BSG (35 kDa), TfR1 (~77.4 kDa), and PHB2 (~35.7 kDa), confirming their purity. The Kaleidoscope molecular weight marker (Bio-Rad) is included in the left lane as a reference.

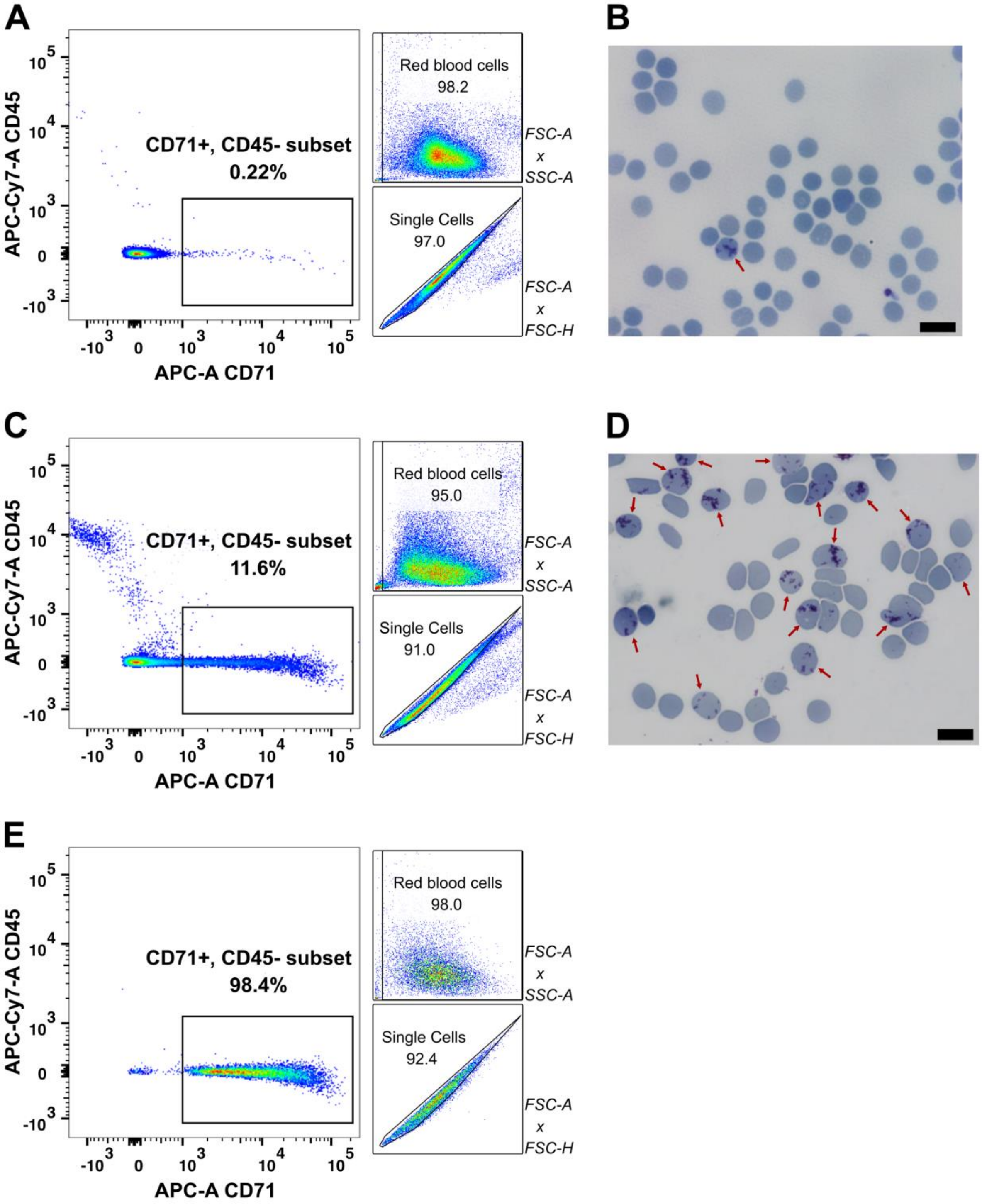

Supplementary Figure 2. Reticulocyte enrichment. **(A, B)** Reticulocyte percentage in the initial whole blood sample. **(C, D)** Reticulocyte percentage after Percoll density (70%-62%) enrichment. **(E)** Purity of reticulocytes ($CD71^+$ $CD45^-$) after sorting collection. This high-purity reticulocyte sample was not evaluated with brilliant cresyl blue due to the small total volume of sample obtained (~ 50 µL). **(B, D)** Blood smears stained with brilliant cresyl blue to identify reticulocytes (red arrows). Scale bars are 10 µm.

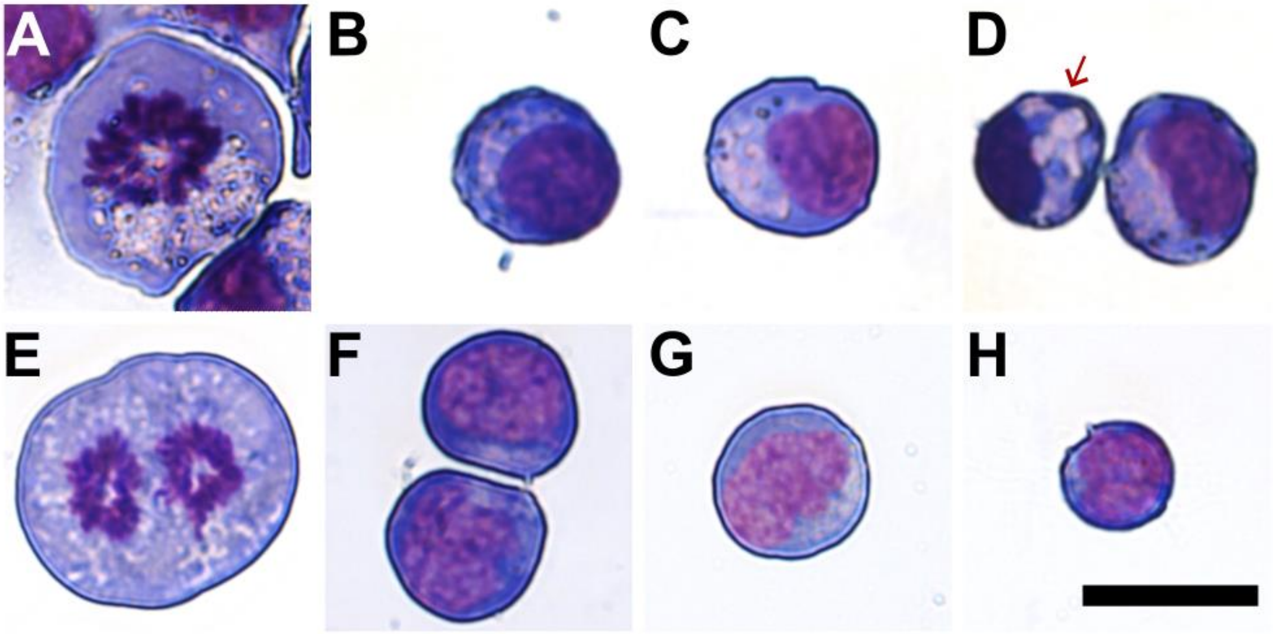

Supplementary Figure 3. Differentiation of erythroid cells. **(A-D)** BEL-A cells. **(E-H)** JK-1 cells. **(A, E)** Proerythroblasts (20-25 µm) were identified by their large nucleus with fine chromatin, prominent nucleoli, and basophilic cytoplasm. These cells progressed to (B, F) Basophilic erythroblasts (16-18 µm), characterized by a slightly smaller nucleus with condensed chromatin and cytoplasm with pronounced basophilia due to increased ribosomes synthesizing Hb. Subsequently, cells differentiated into **(C, G)** polychromatophilic erythroblasts (12-15 µm), which exhibited a more condensed nucleus with clusters of heterochromatin. The final nucleated stage, **(D, H)** orthochromatic erythroblast, indicated by the red arrow in D (10-12 µm), was noted for its pyknotic nucleus and a cytoplasm rich in acidophilic Hb. At this stage, the nucleus is expelled to form reticulocytes (8-10 µm), which then mature into erythrocytes (7-8 µm) through the final step of erythroid differentiation (Yeo et al., 2019).

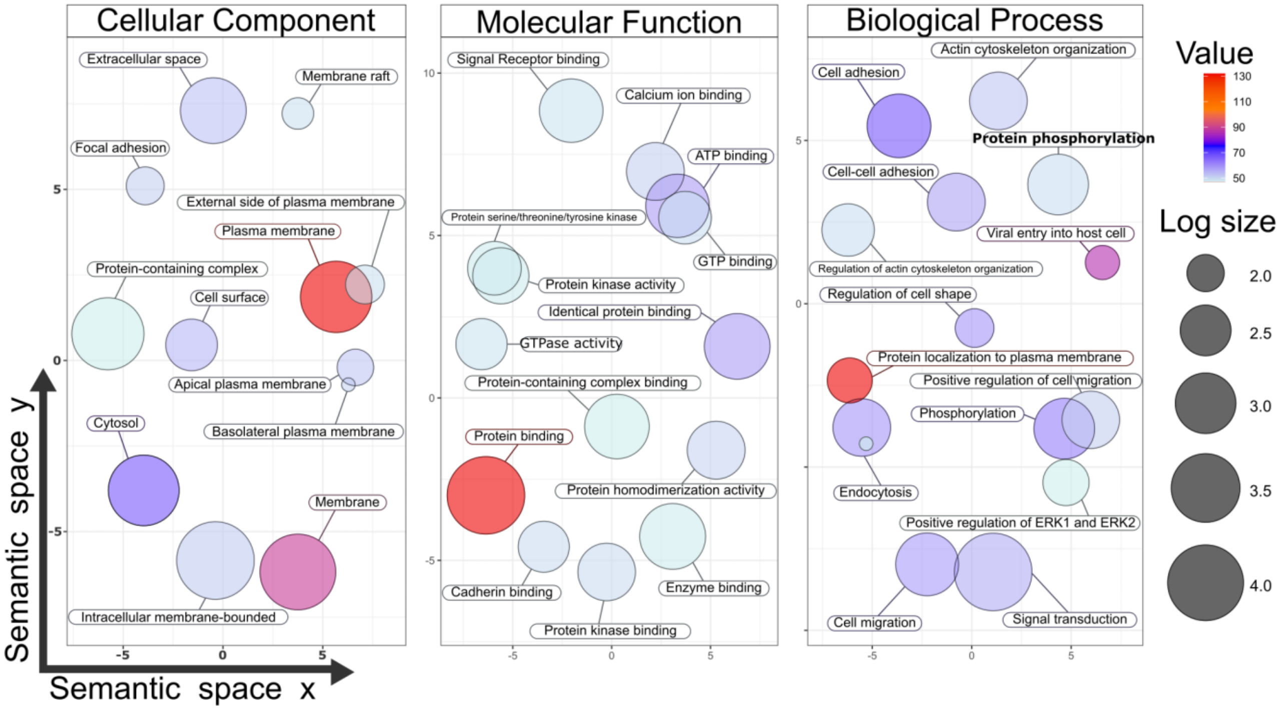

Supplementary Figure 4. Gene Ontology (GO) term analysis of plasma membrane proteins. Scatterplots showing the GO terms: cellular component, molecular function, and biological process of enriched plasma membrane proteins. The bubble color indicates the count of the GO term, which was obtained from the DAVID functional annotation charts (Huang et al., 2009; Sherman et al., 2022). The size of the bubbles indicates the frequency of the GO term in the Gene Ontology Annotation (GOA) database. The axes of the plot have no intrinsic meaning. REVIGO (version 1.8.1) (Supek et al., 2011) was used to generate these scatterplots by reducing the dimensionality of a pairwise semantic similarity matrix of GO terms using Multidimensional Scaling (MDS). The resulting projection is not nonlinear. The guiding principle is that semantically similar GO terms should remain close together in the plot.

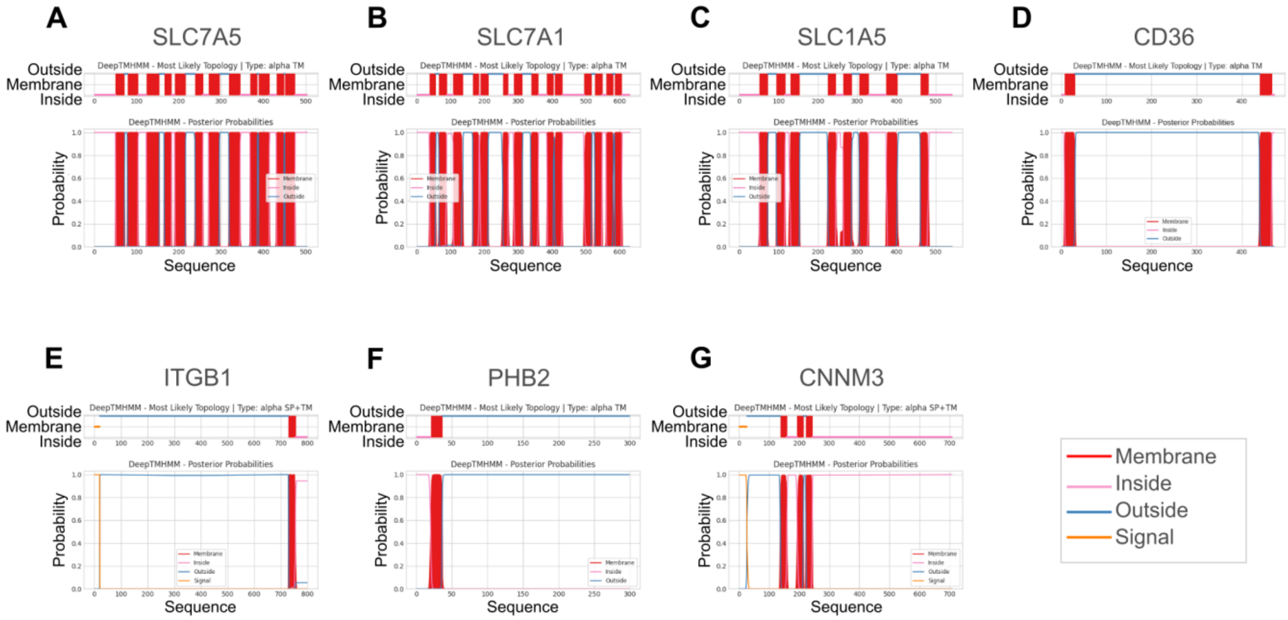

Supplementary Figure 5. Topology of *P. vivax* merozoite receptor candidates. **(A-J)** Predicted locations of potential parasite receptors are illustrated for the signal peptide (yellow), extracellular domain (blue), intracellular domain (mauve), and membrane-embedded domain (red).

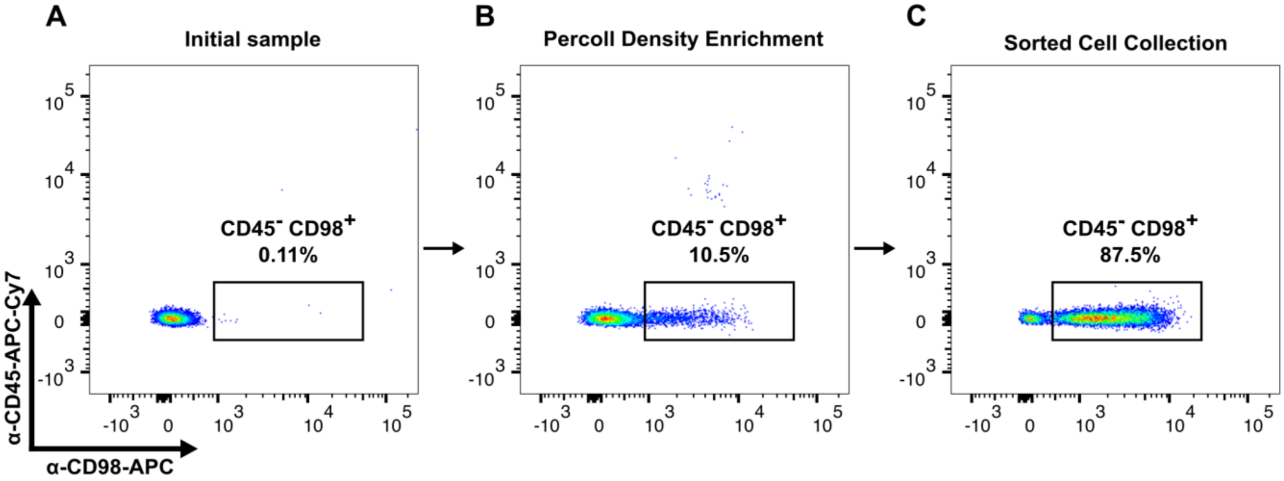

Supplementary Figure 6. Reticulocyte enrichment process. **(A)** Initial percentage of reticulocytes in the whole blood sample (0.11%). **(B)** Percentage of reticulocytes after enrichment using a 70%-62% Percoll density gradient (10.5%). **(C)** Final percentage of reticulocytes ($CD45^-$ and $CD98^+$) following FACS sorting (87.5%).

2.2 Supplementary Table

Supplementary Table 1. *P. vivax* merozoite candidate receptors.

**Gene**	**Protein name**	**Length (aa)**	**TM Helix location**	**Extracellular regions**	**Signal peptide location (Sec/SPI probability)**	**GPI/ non GPI anchor protein Yes/No**	**Ret/Ery**		**BEL-A/Ery**		**JK-1/Ery**	
							**Log2 Fold change**	**Adjusted *P* value**	**Log2 Fold change**	**Adjusted *P* value**	**Log2 Fold change**	**Adjusted *P* value**
SLC7A5	Large neutral amino acids transporter small subunit 1	507	55-75, 83-107, 128-157, 170-186, 195-219, 243-260, 275-299, 323-348, 373-389, 393-417, 435-452, 455-478	76-82, 158-169, 220-242, 300-322, 390-392, 453-454	Non-SP (0.0006)	No	8.06	0.0019	9.06	$7.98E^{-05}$	9.02	0.0001
SLC7A1	High affinity cationic amino acid transporter 1	629	38-55, 66-88, 108-136, 166-183, 188-210, 255-269, 287-311, 338-358, 385-403, 408-429, 495-516, 527-547, 560-579, 585-605	56-65, 137-165, 211-254, 312-337, 404-407, 517-526, 580-584	Non-SP (0.0001)	No	4.09	0.0019	3.03	0.0007	2.33	0.0071
SLC1A5	Neutral amino acid transporter B(0)	541	52-72, 95-116, 131-153, 226-245, 265-285, 306-328, 374-401, 461-481	73-94, 154-225, 286-305, 402-460	Non-SP (0.0002)	No	6.83	0.0023	9.26	$4.34E^{-05}$	8.17	0.0003
CD36	Platelet glycoprotein 4	472	8-30, 440-466	31-439	Non-SP (0)	No	6.57	0.0019	11.30	$2.68E^{-05}$	4.10	0.009
ITGB1	Integrin beta	798	729-754	21-728	1-20 (0.9992)	No	5.13	0.0296	6.62	0.0005	5.71	0.0003
PHB2	Prohibitin-2	299	21-36	37-299	Non-SP (0)	No	8.12	0.0019	6.09	$6.36E^{-05}$	11.05	$4.91E^{-05}$
CNNM3	Metal transporter CNNM3	707	138-158, 193-213, 223-243	26-137, 214-222	1-25 (0.999)	No	5.95	0.0072	4.55	0.0004	8.71	0.0004

3. References

Chambers, M. C., Maclean, B., Burke, R., Amodei, D., Ruderman, D. L., Neumann, S., et al. (2012). A cross-platform toolkit for mass spectrometry and proteomics. *Nat Biotechnol* 30, 918–920. doi: 10.1038/nbt.2377

Demichev, V., Messner, C. B., Vernardis, S. I., Lilley, K. S., and Ralser, M. (2020). DIA-NN: neural networks and interference correction enable deep proteome coverage in high throughput. *Nat Methods* 17, 41–44. doi: 10.1038/s41592-019-0638-x

Huang, D. W., Sherman, B. T., and Lempicki, R. A. (2009). Systematic and integrative analysis of large gene lists using DAVID bioinformatics resources. *Nat Protoc* 4, 44–57. doi: 10.1038/nprot.2008.211

Pino, L. K., Just, S. C., MacCoss, M. J., and Searle, B. C. (2020a). Acquiring and Analyzing Data Independent Acquisition Proteomics Experiments without Spectrum Libraries. *Molecular & Cellular Proteomics* 19, 1088–1103. doi: 10.1074/mcp.P119.001913

Pino, L. K., Searle, B. C., Bollinger, J. G., Nunn, B., MacLean, B., and MacCoss, M. J. (2020b). The Skyline ecosystem: Informatics for quantitative mass spectrometry proteomics. *Mass Spectrometry Reviews* 39, 229–244. doi: 10.1002/mas.21540

Quaglieri, A., Bloom, J., Triantafyllidis, A., Green, B., Condina, M. R., Ngov, P. B., et al. (2022). Mass Dynamics 2.0: An improved modular web-based platform for accelerated proteomics insight generation and decision making. *bioRxiv*, 517480.

R Core Team (2023). *R: A language and environment for statistical computing*. Vienna, Austria: R Foundation for Statistical Computing. Available at: https://www.R-project.org/

Schneider, C. A., Rasband, W. S., and Eliceiri, K. W. (2012). NIH Image to ImageJ: 25 years of image analysis. *Nat Methods* 9, 671–675. doi: 10.1038/nmeth.2089

Sherman, B. T., Hao, M., Qiu, J., Jiao, X., Baseler, M. W., Lane, H. C., et al. (2022). DAVID: a web server for functional enrichment analysis and functional annotation of gene lists (2021 update). *Nucleic Acids Research* 50, W216–W221. doi: 10.1093/nar/gkac194

Snounou, G., Viriyakosol, S., Xin Ping Zhu, Jarra, W., Pinheiro, L., Do Rosario, V. E., et al. (1993). High sensitivity of detection of human malaria parasites by the use of nested polymerase chain reaction. *Molecular and Biochemical Parasitology* 61, 315–320. doi: 10.1016/0166-6851(93)90077-B

Supek, F., Bošnjak, M., Škunca, N., and Šmuc, T. (2011). REVIGO Summarizes and Visualizes Long Lists of Gene Ontology Terms. *PLoS ONE* 6, e21800. doi: 10.1371/journal.pone.0021800

Trakarnsanga, K., Griffiths, R. E., Wilson, M. C., Blair, A., Satchwell, T. J., Meinders, M., et al. (2017). An immortalized adult human erythroid line facilitates sustainable and scalable generation of functional red cells. *Nat Commun* 8, 14750. doi: 10.1038/ncomms14750

Yeo, J. H., Lam, Y. W., and Fraser, S. T. (2019). Cellular dynamics of mammalian red blood cell production in the erythroblastic island niche. *Biophys Rev* 11, 873–894. doi: 10.1007/s12551-019-00579-2

Yu, F., Haynes, S. E., and Nesvizhskii, A. I. (2021). IonQuant Enables Accurate and Sensitive Label-Free Quantification With FDR-Controlled Match-Between-Runs. *Molecular & Cellular Proteomics* 20, 100077. doi: 10.1016/j.mcpro.2021.100077
